# Editorial: Pathogenesis, vaccines, and antivirals against respiratory viruses

**DOI:** 10.3389/fcimb.2023.1202251

**Published:** 2023-05-08

**Authors:** C. Joaquin Caceres, Leonardo Susta, Daniela S. Rajao

**Affiliations:** ^1^ Department of Population Health, College of Veterinary Medicine, University of Georgia, Athens, GA, United States; ^2^ Department of Pathobiology, Ontario Veterinary College, University of Guelph, Ontario, ON, Canada

**Keywords:** viral respiratory diseases, pathogenesis, vaccines & antiviral, zoonotic agents, influenza, SARS-CoV-2

Most respiratory viruses are characterized by high transmissibility, a short incubation period, and mucosal involvement, which makes them less susceptible to the systemic immune response (Clementi et al., 2021; Ursin and Klein, 2021). For these reasons, developing effective countermeasures is often challenging, as exemplified by the pandemic caused by the severe acute respiratory syndrome coronavirus 2 (SARS-CoV-2). Respiratory viruses, such as influenza A and B viruses (FLUAV, FLUBV), human metapneumovirus (hMPV), and respiratory syncytial virus (RSV), are among the leading causes of morbidity and mortality in people worldwide, especially in young children and the elderly (Watson and Wilkinson, 2021; Li et al., 2022; Zimmerman et al., 2022). Other respiratory viruses, such as hantaviruses, can also lead to severe, fatal respiratory disease in humans (Angulo et al., 2015; Liu et al., 2019). Therefore, there is an urgent need to investigate the biology of respiratory viruses in humans and animals relevant to human health to develop more effective mitigating strategies, such as novel vaccine platforms, antiviral compounds, and antibody-based therapies (Yamin et al., 2016; Rajao and Perez, 2018; Caceres et al., 2022). An excellent example of the relevance of understanding the animal-human interface in the context of viral infections is the current FLUAV H5N1 situation, where almost 57 million birds have been affected in the US. Moreover, there is an inherent risk of zoonotic potential associated with these viruses. Different mammalian species, such as raccoons, red foxes, and skunks, have been confirmed positive for H5N1 infections in the US. Human cases have also been reported, highlighting the zoonotic potential of this virus (Prevention CDC, 2023).

In this Research topic, we provided information about the pathogenesis, host-virus interactions, and immune response in the context of infection caused by different respiratory viruses affecting humans and animal species relevant to human health. Furthermore, we provided information about novel vaccines and antiviral therapeutics against other respiratory viruses.

## Original research articles

Oh et al. investigated the potential use of small extracellular vesicles derived from human umbilical cord mesenchymal stem cells (U-exo) against different respiratory viruses such as FLUAV, FLUBV, and human seasonal coronavirus (HCoV) *in vitro.* The treatment of U-exo significantly reduced the replication of H1N1, H3N2, and Yamagata-like FLUBV virus in A549 cells. Further, a synergistic antiviral effect was observed between U-exo and IFN-α in the reduction of viral replication for FLUAV and FLUBV. More in detail, it was demonstrated that miR-125b-5p, one of the highly expressed microRNAs in U-exo, is one of the main responsible for the antiviral activity observed. Similarly, U-exo treatment in HCT-8 cells exerts antiviral activity against HCoV with the same synergic effect previously observed between U-exo and IFN-α. Finally, the authors evaluated the U-exo antiviral activity in a more relevant *in vitro* model using human nasal epithelial cells (HNECs) cultured at the air-liquid interface (ALI). The results revealed an antiviral effect of the U-exo against the different respiratory viruses in this differentiated cell line model.

Li et al explored the SARS-CoV-2 vaccination effect on disease severity and the factors associated with viral clearance and hospitalization, particularly in omicron-infected patients (BA.2). This was carried out during 2 regional outbreaks in China. The results showed a faster transmission, milder symptoms, and lower severity incidence when the omicron group was compared to a group infected with the SARS-CoV-2 delta variant (B.1.167.2). Additionally, a significant inverse correlation of vaccination dose with clinical severity was observed. Moreover, the data revealed that the decrease in disease severity was only significant at ≥21 days after three doses. The data illustrate the differences in the pathogenesis of 2 different SARS-CoV-2 variants of concern (VOCs), such as the delta and omicron, highlighting the benefits of a vaccination schedule, including boost.

## Review articles

Nogales et al. reviewed the current literature regarding the use of NS1 deficient or truncated FLUAV viruses as potential live attenuated influenza vaccines (LAIVs). NS1 is a critical player in the different mechanisms that FLUAV has developed to counteract the host immune. NS1 is a multifunctional protein and virulence factor whose primary role is to balance or modulate host antiviral interferon (IFN) responses at multiple levels. The implementation of reverse genetics for FLUAV has allowed the modification of the FLUAV genome, with the potential of generating novel vaccines for animal or human use. Among the different approaches, truncations or deletions of NS1 have resulted in attenuated viruses, able to elicit a strong immune and adaptive immune response.

Recombinant viruses based on NS1 modifications have been generated and proposed for swine, equine, canine, and avian species. In the case of pigs, attenuation and protection against homo- but alto heterosubtypic isolates were observed in terms of reduced viral shedding after challenge and lower microscopic lung lesions. Moreover, vaccinated analysis was seronegative for NS1, highlighting this approach as an interesting DIVA strategy. A vaccine based on this strategy was the first LAIV licensed in the US for use in pigs in 2017. However, a phylogenetic analysis of whole genome sequences carried out in the US using samples obtained in 2018 indicated that reassortment strains containing LAIV genes in combination with genes from endemic field strains circulating in the US were generated. This suggests a substantial degree of LAIV replication and reassortment between this LAIV and field strains. Because the use of this LAIV interfered with routine swine influenza surveillance in the US, the vaccine was withdrawn from the market in 2020. A similar approach has been evaluated for use against equine Influenza showing that viruses lacking NS1 administered *via* aerosol or intranasal immunization of horses protected against clinical signs upon homologous challenge.

Further, reduced viral replication after the challenge was observed in vaccinated horses. Canine influenza (CIV) has been another target for the development of LAIVs based on this strategy, where results demonstrate NS1 truncated or deficient viruses were attenuated but able to confer complete protection against challenge after a single intranasal immunization in mice. Importantly, immunogenicity and protection efficacy were better than commercial H3N8 CIV-inactivated vaccines.

## Author contributions

All authors listed have made a substantial, direct, and intellectual contribution to the work and approved it for publication.

## References

[B1] Angulo J. Pino K. Echeverria-Chagas N. Marco C. Martinez-Valdebenito C. Galeno H.. (2015). Association of single-nucleotide polymorphisms in IL28B, but not TNF-alpha, with severity of disease caused by Andes virus. Clin. Infect. Dis. 61 (12), e62–e69. 26394672 10.1093/cid/civ830 PMC4657541

[B2] Caceres C. J. Seibert B. Cargnin Faccin F. Cardenas-Garcia S. Rajao D. S. Perez D. R. (2022). Influenza antivirals and animal models. FEBS Open Bio. 12 (6), 1142–1165. doi: 10.1002/2211-5463.13416 PMC9157400 35451200

[B3] Clementi N. Ghosh S. De Santis M. Castelli M. Criscuolo E. Zanoni I.. (2021). Viral respiratory pathogens and lung injury. Clin. Microbiol. Rev. 34 (3). doi: 10.1128/CMR.00103-20 PMC8142519 33789928

[B4] Li Y. Wang X. Blau D. M. Caballero M. T. Feikin D. R. Gill C. J.. (2022). Global, regional, and national disease burden estimates of acute lower respiratory infections due to respiratory syncytial virus in children younger than 5 years in 2019: a systematic analysis. Lancet 399 (10340), 2047–2064. doi: 10.1016/S0140-6736(22)00478-0 35598608 PMC7613574

[B5] Liu R. Ma H. Shu J. Zhang Q. Han M. Liu Z.. (2019). Vaccines and therapeutics against hantaviruses. Front. Microbiol. 10, 2989. doi: 10.3389/fmicb.2019.02989 32082263 PMC7002362

[B6] Prevention CDC (2023). Current U.S. bird flu situation in humans. Available at: https://www.cdc.gov/flu/avianflu/inhumans.htm

[B7] Rajao D. S. Perez D. R. (2018). Universal vaccines and vaccine platforms to protect against influenza viruses in humans and agriculture. Front. Microbiol. 9, 123. doi: 10.3389/fmicb.2018.00123 29467737 PMC5808216

[B8] Ursin R. L. Klein S. L. (2021). Sex differences in respiratory viral pathogenesis and treatments. Annu. Rev. Virol. 8 (1), 393–414. doi: 10.1146/annurev-virology-091919-092720 34081540

[B9] Watson A. Wilkinson T. M. A. (2021). Respiratory viral infections in the elderly. Ther. Adv. Respir. Dis. 15, 1753466621995050. doi: 10.1177/1753466621995050 33749408 PMC7989115

[B10] Yamin D. Jones F. K. DeVincenzo J. P. Gertler S. Kobiler O. Townsend J. P.. (2016). Vaccination strategies against respiratory syncytial virus. Proc. Natl. Acad. Sci. U S A. 113 (46), 13239–13244. doi: 10.1073/pnas.1522597113 27799521 PMC5135296

[B11] Zimmerman R. K. Balasubramani G. K. D’Agostino H. E. A. Clarke L. Yassin M. Middleton D. B.. (2022). Population-based hospitalization burden estimates for respiratory viruses, 2015-2019. Influenza Other Respir. Viruses. 16 (6), 1133–1140. doi: 10.1111/irv.13040 35996836 PMC9530548

